# Development and validation of the Early Pediatric Groningen Defecation and Fecal Continence questionnaire

**DOI:** 10.1007/s00431-022-04714-2

**Published:** 2022-11-21

**Authors:** Sanne J. Verkuijl, Monika Trzpis, Paul M. A. Broens

**Affiliations:** 1 Department of Surgery, Division of Pediatric Surgery, University of Groningen, University Medical Center Groningen, Hanzeplein 1, PO Box 30 001, 9700 RB Groningen, the Netherlands; 2 Department of Surgery, Anorectal Physiology Laboratory, University of Groningen, University Medical Center Groningen, Groningen, the Netherlands

**Keywords:** Infant, Toddler, Gastrointestinal, Incontinence, Constipation

## Abstract

There are no compatible tools that assess bowel function in young children, older children, and adults. This precludes clinical follow-up and longitudinal scientific research. Our aim was therefore to develop and validate a bowel function questionnaire equivalent to the pediatric (8–17 years) and adult (≥ 18 years) Groningen Defecation and Fecal Continence (DeFeC) questionnaires for children from the age of 1 month to 7 years. We developed, validated, and translated the Early Pediatric Groningen DeFeC (EP-DeFeC) questionnaire according to the Consensus-based Standards for the selection of health Measurement Instruments (COSMIN). The EP-DeFeC incorporates different validated bowel function scoring systems, including the Rome IV criteria that are also included in the pediatric and adult DeFeC. We assessed feasibility and reproducibility by a test–retest survey. The study population (*N* = 100) consisted of the parents/caregivers of children whose median age was 4.0 (IQR 2.0–5.0) years. The mean interval between testing and retesting was 2.7 ± 1.1 months. None of the respondents commented on ambiguities regarding the questions. The overall median time taken to complete the EP-DeFeC was 8.7 min (IQR 6.8–11.8). The overall observed agreement was 78.9% with an overall kappa coefficient of 0.51, indicating moderate agreement.

*Conclusion*: The EP-DeFeC is a feasible, reproducible, and validated questionnaire for assessing bowel function in children from the age of 1 month to 7 years. If used in combination with its pediatric (8–17 years) and adult (≥ 18 years) equivalents, this questionnaire enables longitudinal follow-up of bowel function from infancy to adulthood.

**Table:** 

**What is Known:** *• Bowel function problems are common among young children.* *• Unfortunately, there are no compatible tools that assess bowel function in young children, older children, and adults, which precludes clinical follow-up and longitudinal scientific research.*	
**What is New:** *• The Early Pediatric Groningen Defecation and Fecal Continence (EP-DeFeC) questionnaire is validated to assess bowel function in children from the age of 1 month to 7 years.* *• If used together with its pediatric and adult equivalents, longitudinal follow-up of bowel function from infancy to adulthood becomes possible.*

**Supplementary Information:**

The online version contains supplementary material available at 10.1007/s00431-022-04714-2.

## Introduction

Bowel function problems are common among children. In general pediatric populations between 0 and 18 years of age, the prevalence rates for functional constipation vary between 1 and 32% [1]. Estimates of the prevalence of fecal incontinence range between 1 and 4% in toilet-trained children [2]. Recently, extensive community studies showed that no less than 3 to 12% of infants and 10 to 19% of toddlers suffer from functional constipation [3, 4]. In these young children, bowel function problems negatively affect their quality of life and lead to considerable health care costs [4–6]. This underscores the need for standardized diagnostic tools in young children. Nevertheless, many of the existing bowel function questionnaires are unvalidated, address only one aspect of bowel function, can only be used in a specific setting, or they are designed for older children or adults and thereby excluding younger children and infants. Moreover, in order to compare bowel function at different ages, uniform terminology and scores are required [7]. Unfortunately, there are no compatible tools that assess bowel function in young children, as well as in older children and adults. This impairs clinical follow-up of young children with bowel disorders and longitudinal scientific research of bowel function.

The aim of this study was to develop and validate a detailed bowel function questionnaire for children from 1 month to 7 years of age. The questionnaire had to be equivalent to the pediatric (8–17 years) and adult (≥ 18 years) Groningen Defecation and Fecal Continence (DeFeC) questionnaires [8], to enable longitudinal follow-up of bowel function from infancy to adulthood.

## Materials and methods

The questionnaire we developed is equivalent to the Pediatric DeFeC (P-DeFeC) questionnaire for children from 8 to 17 years and the validated adult DeFeC questionnaire for respondents of 18 years and over [8]. We therefore named the newly developed questionnaire the Early Pediatric Groningen Defecation and Fecal Continence (EP-DeFeC) questionnaire. The development, structural validation, and translation of the EP-DeFeC were performed in accordance with the Consensus-based Standards for the selection of health Measurement Instruments (COSMIN) [9, 10]. The study was conducted in compliance with the ethical standards of the Medical Ethical Review Board of University Medical Center Groningen.

### Literature search

We conducted a comprehensive literature search of the PubMed, EMBASE, and Cochrane Library databases to identify the existing bowel function scores and definitions for young children up to 7 years of age. The Medical Subject Heading (MeSH) terms we used were as follows: “Child, Preschool,” “Infant,”, “Surveys and Questionnaires,” “Signs and Symptoms, Digestive,” “Fecal Incontinence,” and “Defecation,” which were supplemented by different keywords.

### Item selection

**Fig. 1:**
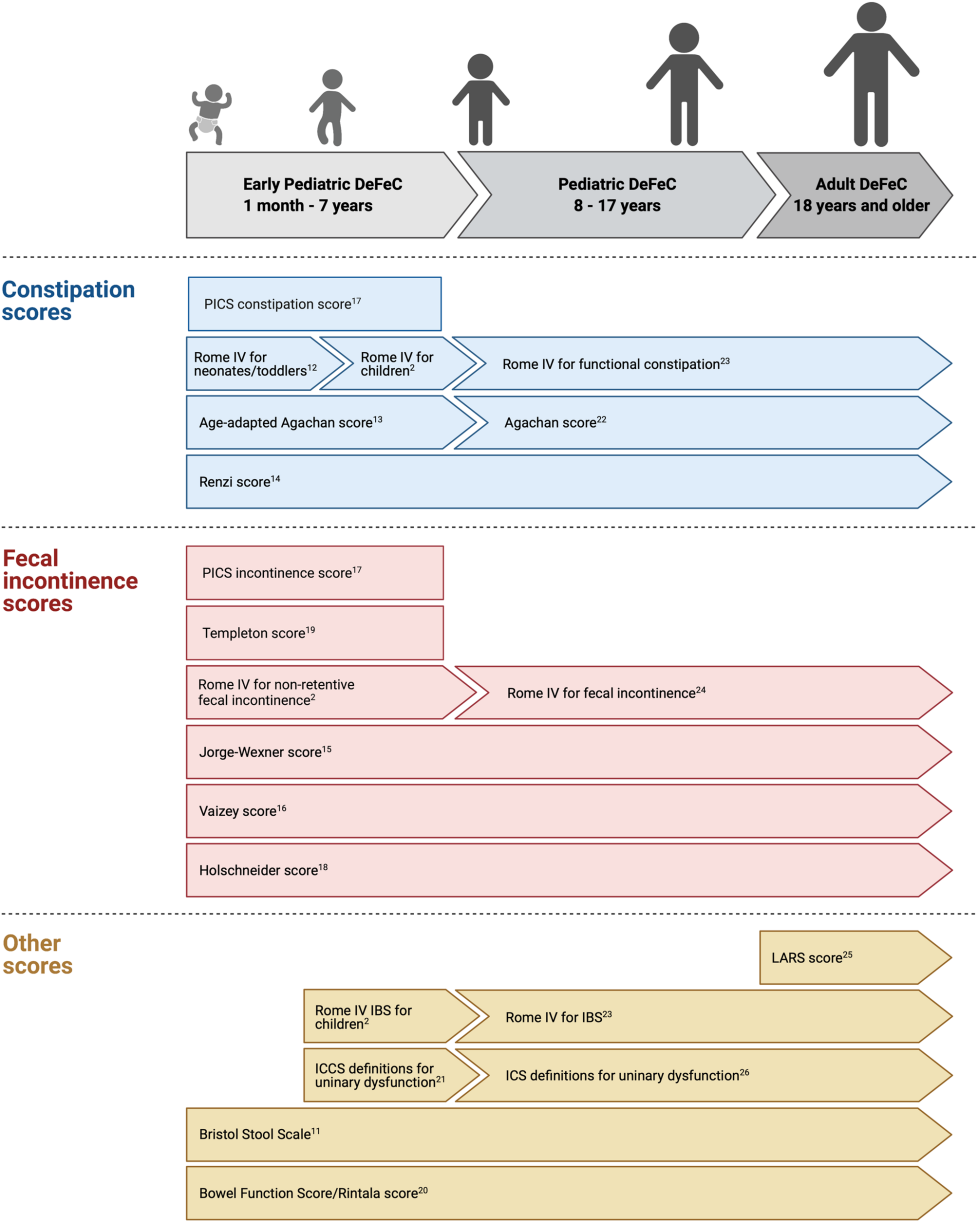
The scoring systems incorporated in all the Groningen Defecation and Fecal Continence (DeFeC) questionnaires. Abbreviations: IBS, irritable bowel syndrome; ICCS, International Children’s Continence Society; ICS, International Continence Society; LARS, low anterior resection syndrome; PICS, pediatric incontinence/constipation score

The items regarding bowel function that we included in the EP-DeFeC are derived from the Bristol Stool Scale [11], the Rome IV criteria for functional constipation in neonates/toddlers [12] and children [2], the Rome IV criteria for irritable bowel syndrome in children [2], the age-adapted Constipation Scoring System [13], the Obstructed Defecation Syndrome score by Renzi and colleagues [14], the Rome IV criteria for non-retentive fecal incontinence in children [2], the Continence Grading Scale by Jorge and Wexner [15], the Vaizey incontinence score [16], the Pediatric Incontinence/Constipation scores [17], the Holschneider score [18], the Templeton score [19], and Rintala and colleagues’ bowel function score [20]. The EP-DeFeC also contains questions concerning urological functioning based on the standardized definitions of the International Children’s Continence Society [21]. Most of these scoring systems, or their adult equivalents [22–26], were also included in the P-DeFeC and the adult DeFeC, which enables the comparison of bowel function from infancy to adulthood (Fig. 1).

On account of the early age of the group targeted for the questionnaire, it was unavoidable that the child’s parents or caregivers completed the EP-DeFeC. Therefore, we adapted all the questions to this setting. We are aware that some of the children can read around the age of 5 or 6 years, but we did not want to introduce a selection bias based on the ability of the child to read. Additionally, we designed the EP-DeFeC in such a way that inapplicable questions could be excluded, for example, the exclusion of questions regarding fecal incontinence if the parents or caregivers had indicated that their child was not yet toilet trained.

In developing the EP-DeFeC, we endeavored to adhere as closely as possible to the format of the existing P-DeFeC and adult DeFeC questionnaires (Table 1) [8]. Thus, the EP-DeFeC consisted of eight categories: personal details, defecation pattern, constipation, constipation-related questions, fecal continence, urge, urinary continence, and medical history. We did not include questions from the P-DeFeC and adult DeFeC that did not apply to children between 1 month and 7 years of age, such as residential information, educational level, work, and obstetric/gynecological medical history. On the contrary, we added additional age-related questions to the EP-DeFeC, such as a preterm birth and type of feeding (Table 1).

**Table 1:** Categories and questions of all the Groningen Defecation and Fecal Continence (DeFeC) questionnaires

	**Early Pediatric DeFeC**		**Pediatric DeFeC**		**Adult DeFeC**	
	**1 month–7 years**		**8–17 years**		** ≥ 18 years**	
**Categories** **and number of questions**	Personal details	3	Personal details	5	Personal details	8
	Defecation pattern	4	Defecation pattern	2	Defecation pattern	2
	Constipation	14	Constipation	16	Constipation	16
	Constipation-related questions	16	Constipation-related questions	14	Constipation-related questions	14
	Fecal continence	17	Fecal continence	16	Fecal continence	16
	Urge	3	Urge	4	Urge	4
	Urinary continence	10	Urinary continence	9	Urinary continence	9
					Obstetric and gynecological history	11
	Medical history	8	Medical history	8	Medical history	8
	**Total**	**75**	**Total**	**74**	**Total**	**88**
**Additional** **age-specific questions**	Preterm birth, type of feeding (breast milk/formula/solids), recent dietary changes, toilet training		Residence		Residence, educational level, profession, working life	

### Pilot testing

We sent the first draft of the EP-DeFeC to a Delphi panel consisting of pediatricians, pediatric surgeons, pediatric gastroenterologists, pediatric physiotherapists, and specialized pediatric nurses. They were specifically asked to provide feedback on completeness, relevance, redundancy, and/or the wording of the questions and to test the content validity of the questionnaire [9, 10]. All remarks were discussed and incorporated in the second draft of the EP-DeFeC.

### Feasibility

The second draft of the EP-DeFeC was distributed randomly among native Dutch-speaking parents with various levels of education who had children aged between 1 month and 7 years. The parents were asked to read the questionnaire thoroughly and to mention any ambiguous questions or answer options. After the pilot testing was completed, the problems raised by the parents were discussed and revised. This resulted in the definitive version of the EP-DeFeC, which consisted of 75 questions (Online Resource 1).

### Reproducibility and reliability

We performed a test–retest survey to determine the reproducibility of all questions and the reliability of the different scoring systems in the EP-DeFeC. We invited a randomly selected sample of Dutch parents of children between 1 month and 7 years of age to complete the EP-DeFeC. All participants were recruited by an external survey company (Dynata, Rotterdam, the Netherlands) from all regions of the Netherlands. Respondents were asked to complete the EP-DeFeC again after approximately 3 months. This time interval was chosen to ensure that the test–retest interval was long enough to prevent recall but short enough that the likelihood of major changes in the respondent’s circumstances was minimal, according to the COSMIN principles [9]. The respondents were not initially informed about the fact that they would be asked to complete the questionnaire again. We also assessed how long it took to complete the questionnaire. We excluded respondents who reported a different date of birth and/or sex of their child on the test and retest forms, which rendered these questionnaires invalid. All questionnaires were completed digitally, with the obligation to complete all applicable questions before submitting the questionnaire.

### Translation

The final EP-DeFeC was translated from Dutch into English. In accordance with the COSMIN principles, the translation was performed by two independent professional translators who were naive as to the topic [9]. One translator translated the questionnaire into English and the other translated the questionnaire back into Dutch. Discrepancies between the translations were discussed and resolved in the final English version of the EP-DeFeC.

### Statistical analysis

Descriptive statistics on personal characteristics and the time required to complete the questionnaire are shown as mean ± SD or median (IQR), depending on their distribution. Categorical variables are shown as number (percentage). We calculated the percentage of observed agreement for all categorical and dichotomous questions. We evaluated the reproducibility of all questions by calculating the unweighted kappa coefficient for the questions with dichotomous answer options and the weighted Cohen kappa coefficient for the questions with multiple, ordinal answer options. To examine the reproducibility of the scoring systems, the unweighted kappa coefficient was determined for the scoring systems with a dichotomous outcome. The reliability of the continuous scoring systems was expressed as intraclass correlation coefficient (ICC). All kappa coefficients and ICC values were interpreted according to Landis and Koch—values between 0.01 and 0.20 were considered slight agreement, values between 0.21 and 0.40 as fair agreement, values between 0.41 and 0.60 as moderate agreement, values between 0.61 and 0.80 as substantial agreement, and values between 0.81 and 1.00 as almost perfect agreement [27]. All analyses were performed with SPSS software, Version 23.0 (Armonk, NY, USA: IBM Corp), and the image was created with Biorender.com.

## Results

A total of 124 parents of children between 1 month and 7 years of age completed the test and retest of the EP-DeFeC without any missing values. We excluded 24 of these respondents because the date of birth and/or sex of the child between the test and retest did not match. The children of the 100 included respondents had a median age of 4.0 (IQR 2.0–5.0) years, and 57% of them were boys. The mean time interval between the test and retest was 2.7 ± 1.1 months. The mean time interval between the test and retest was not significantly different between infants/toddlers and older children (2.5 versus 2.9 months, *p* = 0.092).

### Feasibility

None of the respondents commented on ambiguities regarding any of the 75 questions of the final EP-DeFeC. The overall median time to complete the EP-DeFeC was 8.7 min (IQR 6.8–11.8, Table 2). Parents of a child who always wore a diaper took a median of 7.9 (IQR 5.9–9.9) minutes to complete the EP-DeFeC. Parents of a child who had just started toilet training or who was fully toilet trained spent a median of 9.5 (IQR 8.8–13.5) and 9.0 (IQR 7.3–12.2) minutes, respectively, to complete the EP-DeFeC (Table 2).

**Table 2:** Applicable questions and the time required to complete the Early Pediatric Groningen Defecation and Fecal Continence (EP-DeFeC) questionnaire in three different settings

**Category**	**No. of questions**	**Topic of the questions**	**Setting 1**	**Setting 2**	**Setting 3**
			**Child always wears a diaper**	**Child has started** **toilet training**	**Child is fully** **toilet trained**
Personal details	3	General information such as sex and preterm birth	3	3	3
Defecation pattern	4	Defecation frequency and stool consistency	4	4	4
Constipation	14	Difficulties passing stool, failure to defecate, and anal or abdominal pain	14	14	14
Constipation-related questions	16	Diet, laxatives, and/or more invasive therapies for constipation	12	16	16
Fecal continence	17	Toilet training for feces, different types of fecal incontinence, and/or therapies for incontinence	1	17	17
Urge	3	Urge to defecate, ability to hold stool, ability to differentiate	3	3	3
Urinary continence	10	Toilet training for urine, different types of urinary incontinence, and urinary tract infections	2	10	10
Medical history	8	History of gastrointestinal surgery, presence of blood or slime in stools, medical conditions affecting bowel movements, and overall medication use	8	8	8
**Total number of questions**	**75**		**47**	**75**	**75**
**Time to complete the questionnaire (minutes)**^**a**^	**8.7** **(6.8–11.8)**		**7.9** **(5.9–9.9)**	**9.5** **(8.8–13.5)**	**9.0** **(7.3–12.2)**

^a^Values are presented as medians with interquartile ranges

### Reproducibility of all questions of the EP-DeFeC

The percentage of observed agreement of the questions in every category of the EP-DeFeC ranged between 67.7 and 91.8%, with mean kappa coefficients between 0.38 and 0.60 (Table 3). All categories except defecation pattern showed moderate or substantial agreement. The overall observed agreement of the EP-DeFeC was 78.9%, with an overall kappa coefficient of 0.51, indicating moderate agreement (Table 3). The overall observed agreement and overall kappa coefficient were not different between infants/toddlers and older children (Online Resource 2).

**Table 3:** Reproducibility of the Early Pediatric Groningen Defecation and Fecal Continence (EP-DeFeC) questionnaire

**Category**	**Observed agreement (%)**	**Kappa coefficient**	**Interpretation**^**a**^
Defecation pattern	72.5	0.38	Fair
Constipation	75.2	0.43	Moderate
Constipation-related questions	91.8	0.53	Moderate
Fecal continence	77.6	0.53	Moderate
Urge to defecate	67.7	0.60	Moderate
Urinary continence	78.4	0.57	Moderate
Medical history	88.8	0.52	Moderate
**Overall**	**78.9**	**0.51**	**Moderate**

^a^Interpretation of kappa coefficients according to Landis and Koch^27^

### Reliability of the incorporated scoring systems of the EP-DeFeC

The kappa coefficients of the dichotomous scores and the intraclass correlation coefficients of the continuous scores ranged between 0.34 and 0.91 (Table 4). The majority of the incorporated scoring systems showed almost perfect agreement. The percentage of observed agreement of the continuous scores ranged between 89.1 and 96.4% (Table 4A). The unweighted kappa coefficient for the Rome IV criteria of functional constipation for neonates/toddlers was 0.65 (95% CI, 0.20–1.10), for the Rome IV criteria of functional constipation for children 0.47 (95% CI, 0.05–0.89), for the Rome IV criteria for irritable bowel syndrome for children 0.85 (95% CI, 0.65–1.05), and for the Rome IV criteria for non-retentive fecal incontinence for children 0.34 (95% CI, − 0.07–0.75, Table 4A). The mean intraclass correlation coefficient for all continuous scores for fecal incontinence was 0.87, including the Continence Grading Scale, the Vaizey Continence score, the Holschneider score, the Templeton score, and the PICS incontinence score (Table 4B). This indicates an almost perfect overall agreement for the continuous scores for fecal incontinence. For the continuous scores for constipation, the mean intraclass correlation coefficient was 0.71, including the Constipation Scoring System, the Renzi score, and the PICS constipation score (Table 4B). This indicates a substantial overall agreement for the continuous scores for constipation.

**Table 4:** Reliability of the incorporated bowel function scores of the Early Pediatric Groningen Defecation and Fecal Continence (EP-DeFeC) questionnaire

A. **Dichotomous scores**				
	**Observed agreement (%)**	**Unweighted kappa coefficient**	**95% CI**	**Interpretation**^a^
Rome IV for functional constipation, 0–3 years^12^	95.6	0.65	0.20–1.10	Substantial
Rome IV for functional constipation, 4–7 years^2^	92.7	0.47	0.05–0.89	Moderate
Rome IV for IBS, 4–7 years^2^	96.4	0.85	0.65–1.05	Almost perfect
Rome IV for non-retentive fecal incontinence, 4–7 years^2^	89.1	0.34	− 0.07–0.75	Fair
B. **Continuous scores**				
	**Intraclass correlation coefficient (ICC)**		**95% CI**	**Interpretation**^a^
PICS constipation score^17^	0.66		0.53–0.76	Substantial
Age-adapted Agachan score^13^	0.80		0.72–0.86	Substantial
Renzi score^14^	0.67		0.54–0.76	Substantial
PICS incontinence score^17^	0.88		0.83–0.92	Almost perfect
Templeton score^19^	0.84		0.77–0.89	Almost perfect
Jorge-Wexner score^15^	0.85		0.79–0.90	Almost perfect
Vaizey score^16^	0.86		0.79–0.90	Almost perfect
Holschneider score^18^	0.91		0.87–0.94	Almost perfect
Bristol Stool Scale^11^	0.50		0.34–0.63	Moderate
Bowel function score/Rintala score^20^	0.85		0.79–0.90	Almost perfect

*IBS* irritable bowel syndrome, *PICS* pediatric incontinence/constipation score

^a^Interpretation according to Landis and Koch^27^

## Discussion

We developed the EP-DeFeC questionnaire to assess the bowel function of children between 1 month and 7 years of age (Online Resource 1). Overall, the EP-DeFeC was found to be reproducible and structurally valid in Dutch children of this age.

The EP-DeFeC incorporates various existing, well-known bowel function scores and definitions, such as the Rome IV criteria for functional constipation, the PICS incontinence and constipation scores, the Jorge-Wexner score, and many others (Fig. 1) [2, 11–20]. Both constipation and fecal incontinence are addressed by the EP-DeFeC as these conditions often go together in children [17, 28]. The bowel function scores facilitate the clinical interpretation of the questionnaire, because only counting fecal incontinence episodes or knowing stool frequency does not necessarily differentiate between children with acceptable bowel function and those in need of medical attention. Moreover, the incorporated validated bowel function scores and definitions for constipation and fecal incontinence also benefit the application of the EP-DeFeC for scientific research purposes. The scientific use of the EP-DeFeC is also supported by the fact that the questionnaire incorporates almost all aspects of the core outcome set for childhood constipation [29].

Most of the incorporated bowel function scores [2, 11–21], or their adult equivalents [22–24], are also present in the P-DeFeC and adult DeFeC. Therefore, subsequent use of the EP-DeFeC (1 month to 7 years), P-DeFeC (8 to 17 years), and adult DeFeC (18 years and over) enables follow-up of bowel function from infancy to adulthood. To the best of our knowledge, the series of DeFeC questionnaires is the first tool developed specifically to determine the course of bowel function problems from infancy to adulthood effectively, using validated scoring systems and definitions. The follow-up of bowel function over time will add to our knowledge of the physiological development of bowel function from early childhood to adulthood. Furthermore, the tool can be used to illustrate the course of distinct aspects of the bowel function during the clinical follow-up of young children with bowel disorders, such as Hirschsprung disease and congenital anorectal malformations.

Although bowel function is the main topic of the EP-DeFeC, additional questions about sex, preterm birth, diet, therapies, medical history, and familial medical history are included. Previously, these factors were associated with bowel function [1, 3, 5, 30, 31]. Furthermore, the EP-DeFeC also includes urological questions, because it is known that children often suffer from concomitant bladder and bowel dysfunction [32]. These additional questions make the EP-DeFeC a feasible tool to screen for coexisting and/or causative factors of the detected bowel function problems. Therefore, the answers provided in the EP-DeFeC questionnaire may indicate the need for drug therapy, dietary advice, and/or bowel training programs.

The development and testing of the EP-DeFeC among a diverse sample of respondents adds to its applicability. We distributed the questionnaire among Dutch parents with young children from all regions within the Netherlands and with various educational levels. Furthermore, the feasibility analysis shows that the EP-DeFeC is usually quick and easy to complete. The fact that inapplicable parts of the EP-DeFeC can be excluded may have added to its feasibility in this young age group with a large physiological variation in bowel function. Hopefully, the short time to complete the EP-DeFeC may stimulate its use prior to appointments with a health care provider, at home, or even in the waiting room. Additionally, the EP-DeFeC has been officially translated from Dutch into English and German, making it readily available for international use.

The results show a moderate overall agreement between the test and retest of the EP-DeFeC, which corroborates with the overall reproducibility of the adult DeFeC [8]. The reliability of the Rome IV criteria within the EP-DeFeC was comparable to previous reports [33]. Nevertheless, there was a remarkable difference between the high percentages of observed agreement of some questions and low kappa coefficients. This is mainly caused by extremely low kappa coefficients for questions with small numbers of positive answers, a statistical phenomenon which is termed the Cicchetti paradox [34]. In this case, the low kappa values reflect a low prevalence of the symptom in the cohort instead of a lack of reproducibility. Therefore, we provided both the observed agreement and the kappa coefficients.

An important strength of the present study is that the EP-DeFeC was developed and validated according to the structured COSMIN methods [9, 10]. The main limitation of the current study is that we could not determine the diagnostic validity of the EP-DeFeC, because currently no gold standard exists to objectively assess constipation and/or fecal incontinence in young children because both conditions are symptom-based diagnoses. Furthermore, the EP-DeFeC is created as a quick and easy tool to screen for bowel problems, without requiring physical examination. The Rome IV criterion of presence of a large fecal mass in the rectum, which points to functional constipation, was therefore not taken into account [2, 12]. In this way, the EP-DeFeC may slightly underestimate the prevalence of constipation, although it has been reported that digital rectal examination only has a marginal added value in satisfying the Rome criteria for functional constipation [35]. Another limitation is that the incorporated bowel function scores are not all validated for use in young children. Lastly, the assessment of fecal incontinence in young children is particularly challenging because it depends not only on disease severity, but also on the physiological ability to be toilet trained at a certain age. Therefore, it is important to keep the age of the child and the toilet training status in mind while interpreting the scores for fecal incontinence.

In conclusion, the EP-DeFeC is a feasible and validated questionnaire for assessing bowel function, coexisting disorders, and/or causative factors in children from 1 month to 7 years of age. The development of this questionnaire enables longitudinal follow-up of bowel function from infancy to adulthood, when combined with its pediatric (8 to 17 years) and adult (18 years and over) equivalents. We therefore encourage using the EP-DeFeC for longitudinal clinical purposes and for scientific research.

## Supplementary Information

Below is the link to the electronic supplementary material.

Supplementary file1 (PDF 469 KB)

Supplementary file2 (PDF 91 KB)

## Abbreviations

COSMIN: Consensus-based Standards for the selection of health Measurement Instruments
DeFeC: Defecation and Fecal Continence
EP-DeFeC: Early Pediatric DeFeC
IBS: Irritable bowel syndrome
ICC: Intraclass correlation coefficient
ICCS: International Children’s Continence Society
ICS: International Continence Society
LARS: Low anterior resection syndrome
MeSH: Medical Subject Heading
P-DeFeC: Pediatric DeFeC
PICS: Pediatric incontinence/constipation score
