# Supplementary material for: Development and validation of the Early Pediatric Groningen Defecation and Fecal Continence questionnaire
Source: Eur J Pediatr. 2022 Nov 21;182(2):615–23. doi: 10.1007/s00431-022-04714-2 (PMC9899161; doi:10.1007/s00431-022-04714-2)
Supplement: Supplementary file 1 — Supplementary file1 (PDF 469 KB) [file 431_2022_4714_MOESM1_ESM.pdf]

The early pediatric Groningen
Defecation & Faecal Continence Questionnaire

For children aged 1 month to 7 years

Instructions:

1. This questionnaire contains questions about your child that we would like you, in your role as a parent or caregiver, to answer.
2. Answer the questions by checking the relevant box. For each question, select one answer (unless it is specifically indicated that multiple answers are possible).
3. Some questions may look similar, but they all provide us with important information. Some questions may additionally refer to problems that your child does not have. The answers to these questions also provide us with important information. We therefore ask you to answer all questions (unless it is specifically indicated that you can skip questions).
4. There are no right or wrong answers. If you are unsure about the answer, select the answer that most closely approximates your situation.
5. If you have any remarks about the questionnaire, or if you wish to share information that is not asked, please use the space at the end of the questionnaire.
6. Your answers will be treated as strictly confidential.

umcg

Personal details

Date on which you completed this questionnaire ____________________

Your child's last name ____________________

Your child's first name ____________________

Your child's date of birth ____________________

Your child's current height (cm) ____________________

Your child's current weight (kg) ____________________

0.1 What is your child's sex?

- ☐ Male
- ☐ Female

0.2 Was your child born before the 37th week of pregnancy (prematurely)?

- ☐ No, my child was born on time (37 weeks or later)
- ☐ Yes, my child was born prematurely

  The birth took place in week ___________ of the pregnancy

0.3 How would you describe your child's health with respect to his/her ability to hold and pass stool?

- ☐ Very good
- ☐ Good
- ☐ Reasonable
- ☐ Poor
- ☐ Very poor

Category 1: Defecation pattern

The following questions concern your child's defecation pattern <u>in the past month</u>

1.1 On average, how often did your child pass stool? (**Select one answer**)

- ☐ Less than once a month
- ☐ Less than once a week
- ☐ Once a week
- ☐ Twice a week
- ☐ Once every two days
- ☐ Once a day
- ☐ Twice a day
- ☐ Three to five times a day
- ☐ More than five times a day

1.2 How did your child’s stools generally look? **(Select one answer)**

- ☐ 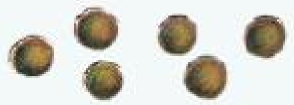 Separate hard lumps (hard to pass)
- ☐ 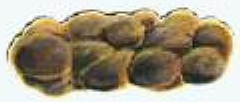 Sausage-shaped but lumpy
- ☐ 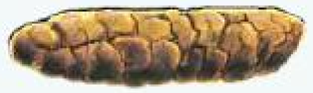 Like a sausage but with cracks on its surface
- ☐ 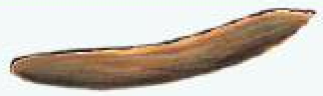 Like a sausage or snake, smooth and soft
- ☐ 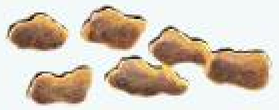 Soft blobs with clear-cut edges (passed easily)
- ☐ 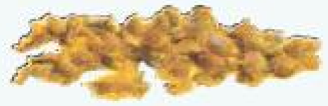 Fluffy pieces with ragged edges, a mushy stool
- ☐ 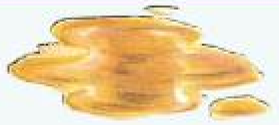 Watery, no solid pieces (enterily liquid)

1.3 Did your child’s stools vary much in terms of hardness?

- ☐ No, they are usually the same
- ☐ Yes, they are different every day

1.4 Did your child’s stools have a large diameter? (For example, were they sometimes difficult to flush through the toilet?)

- ☐ No
- ☐ Yes

Category 2: Constipation

The following questions concern any difficulties your child may have experienced in passing stool <u>in the past month</u>

2.1 How often did your child experience difficulties passing stool (constipation)?

- ☐ Never
- ☐ Less than once a month
- ☐ Several times a month
- ☐ Several times a week
- ☐ Every day

2.1.1 If so, how long has your child had difficulties passing stool (constipation)?

- ☐ 0-1 year
- ☐ 1-3 years
- ☐ 3-5 years
- ☐ 5-7 years

2.2 How often did your child have to strain to pass stool?

- ☐ Never
- ☐ Less than once a month
- ☐ Several times a month
- ☐ Several times a week
- ☐ Every day

2.3 How long did your child generally need to pass stool?

- ☐ Less than 5 minutes
- ☐ 5 to 10 minutes
- ☐ 10 to 20 minutes
- ☐ 20 to 30 minutes
- ☐ Longer than 30 minutes

2.4 Did it happen that despite straining long and hard your child could only pass a small amount of stool?

- ☐ Never
- ☐ Less than once a month
- ☐ Several times a month
- ☐ Several times a week
- ☐ Every day

2.5 How often was your child unable to pass any stool, despite indicating that he/she needed to go to the toilet or was straining?

- ☐ He/she was always able to pass stool
- ☐ One to three times a day
- ☐ Four to six times a day
- ☐ Seven to nine times a day
- ☐ More than nine times a day

2.6 How often did your child experience pain when passing stool?

- ☐ Never
- ☐ Less than once a month
- ☐ Several times a month
- ☐ Several times a week
- ☐ Every day

2.7 How often did your child hold their stool **on purpose** when you were at home and there was a toilet in the vicinity?
(Some children do this, for example, by crossing their legs or sitting or standing with their legs held stiff)

- ☐ Never
- ☐ Less than once a month
- ☐ Several times a month
- ☐ Several times a week
- ☐ Every day

2.8 When did your child pass stool?

- ☐ At different times during the day, without any clear preference
- ☐ Only at moments of complete relaxation (for example while sleeping, in the bath or in the shower)

2.9 Did your child pass gas a lot?

- ☐ Never
- ☐ Sometimes
- ☐ Always

2.10 Did your child have abdominal pain or cramps (tummy ache)?

- ☐ Never
- ☐ Less than once a month
- ☐ Several times a month
- ☐ Several times a week
- ☐ Every day

If your child did not have abdominal pain or cramps in the last month, please proceed to question 3.1.

2.10.1 When your child had abdominal pain or cramps, did these symptoms disappear or improve after your child passed stool?

- ☐ Never or rarely
- ☐ Sometimes
- ☐ Often
- ☐ Usually
- ☐ Always

2.10.2 Did your child pass stool more or less often when he/she had abdominal pain or cramps?

- ☐ Yes, my child passed stool more often at such times
- ☐ Yes, my child passed stool less often at such times
- ☐ No, my child passed stool as frequently as at other times

2.10.3 Did your child's stools look different when he/she had abdominal pain or cramps?

- ☐ Yes, my child's stools were harder at such times
- ☐ Yes, my child's stools were softer at such times
- ☐ No, my child's stools looked the same

Category 3: Constipation-related questions

The following questions concern nutrition and potential remedies that your child may have used to help him/her pass stool <u>in the past month</u>

3.1 Did your child have difficulty drinking enough (compared to other children of the same age)?

- ☐ Yes
- ☐ No

3.2 What did your child's diet consist of?

- ☐ Only breast milk
- ☐ Only formula milk
- ☐ A mix of breast milk and formula milk
- ☐ Solids as well as breast milk and/or formula milk
- ☐ My child ate the same food as the rest of the family

If your child only drank milk, please proceed to question 3.7

3.3 Did your child eat fruit every day?

- ☐ Yes
- ☐ No

3.4 Did your child eat vegetables every day?

- ☐ Yes
- ☐ No

3.5 Did your child eat wholemeal porridge or brown or wholemeal bread?

- ☐ Yes
- ☐ No

3.6 Did your child's diet change in the past month?

- ☐ No
- ☐ Yes, my child recently started eating solids in addition to milk
- ☐ Yes, other ___________________

3.7 How often was your child given a laxative to make his/her stool softer?

- ☐ Never
- ☐ Less than once a month
- ☐ Several times a month
- ☐ Several times a week
- ☐ Every day
- ☐ Several times a day

3.7.1 If your child was given a laxative, what medicine was this and in what dose?

1. Medicine: ________________	How many times a day: ____<br>Or, how many times a week: ____	Dose: ____ ml/g
2. Medicine: ________________	How many times a day: ____<br>Or, how many times a week: ____	Dose: ____ ml/g
3. Medicine: ________________	How many times a day: ____<br>Or, how many times a week: ____	Dose: ____ ml/g

3.8 Was your child on a special diet or did he/she eat special foods to make his/her stool softer?

- ☐ Yes, my child eats/drinks: ____________________
- ☐ No

3.9 Was your child given an enema (= small quantity of medicine inserted into the anus) to help him/her pass stool?

- ☐ Yes, medicine: ____________________ Dose: _____ ml/cc
- ☐ No

3.9.1 If so, how often?

- ☐ Less than once a month
- ☐ Several times a month
- ☐ Several times a week
- ☐ Every day
- ☐ Several times a day

3.10 Did you rinse your child's colon with lukewarm water or a saline solution to help him/her pass stool (via the anus or a stoma)?

- ☐ Yes, quantity: _____ ml/cc, any additions: ____________
- ☐ No

3.10.1 If so, how often?

- ☐ Less than once a month
- ☐ Several times a month
- ☐ Several times a week
- ☐ Every day
- ☐ Several times a day

3.11 Did you sometimes help your child pass stool using your fingers or hands?

- ☐ Yes, by pressing with my hands on his/her abdomen
- ☐ Yes, by pressing between the buttocks, just <u>in front of</u> the anus
- ☐ Yes, by pressing between the buttocks, just <u>behind</u> the anus
- ☐ Yes, by removing the stool from the anus with my fingers
- ☐ Yes, in some other way, namely: ___________________
- ☐ No

3.11.1 If so, how often did you help your child pass stool using your fingers or hands?

- ☐ Less than once a month
- ☐ Several times a month
- ☐ Several times a week
- ☐ Every day

3.12 If your child had difficulties passing stool, did you ever speak about this with someone else? (Multiple answers possible)

- ☐ Not applicable; my child has no difficulties passing stool
- ☐ Yes, with relatives or friends
- ☐ Yes, with the child healthcare centre
- ☐ Yes, with the GP
- ☐ Yes, with a medical specialist
- ☐ Yes, with someone else, namely: ___________________
- ☐ No

Category 4: Faecal continence

The following questions concern your child's faecal <u>continence in the past month</u>

4.1 Which of the following most closely describes your child's situation (**select one answer**)?

- ☐ My child is toilet trained for stool
- ☐ My child is being toilet trained for stool
- ☐ My child has not yet started toilet training for stool and still wears a nappy **<u>at all times</u>**

If your child has not yet started toilet training for stool and wears a nappy at all times, please proceed to question 5.1

4.2 How often did your child accidentally pass small quantities of stool (for example soiling his/her underwear)?

- ☐ Never
- ☐ Less than once a month
- ☐ Several times a month
- ☐ Several times a week
- ☐ Once a week
- ☐ Every day
- ☐ Several times a day

4.3 How often did your child accidentally pass large quantities of stool (for example, leading to all his/her clothes having to be changed)?

- ☐ Never
- ☐ Less than once a month
- ☐ Several times a month
- ☐ Several times a week
- ☐ Every day
- ☐ Several times a day

4.4 How often did your child indicate the need to defecate, but was unable to reach the toilet in time?

- ☐ Never
- ☐ Less than once a month
- ☐ Several times a month
- ☐ Several times a week
- ☐ Every day
- ☐ Several times a day

4.5 How often did your child accidentally pass liquid stool (diarrhoea)?

- ☐ Never
- ☐ Less than once a month
- ☐ Several times a month
- ☐ Several times a week
- ☐ Every day
- ☐ Several times a day

4.6 How often did your child accidentally pass gas?

- ☐ Never
- ☐ Less than once a month
- ☐ Several times a month
- ☐ Several times a week
- ☐ Every day
- ☐ Several times a day

If your child did not accidentally pass liquid or solid stool in the past month, please proceed to question 5.1

4.7 Did your child wear a nappy **during the day**?

- ☐ Never
- ☐ Sometimes
- ☐ Always

4.8 Did your child wear a nappy **at night**?

- ☐ Never
- ☐ Sometimes
- ☐ Always

4.9 How often did your child need a spare set of underwear or a panty liner because he/she may accidentally pass stool?

- ☐ Never
- ☐ Only when my child had diarrhoea
- ☐ Less than once a month
- ☐ Several times a month
- ☐ Several times a week
- ☐ Every day
- ☐ Several times a day

4.10 How often did you have to adjust your child's activities because he/she may accidentally pass stool (for example, avoid swimming or playing sports)?

- ☐ Never
- ☐ Less than once a month
- ☐ Several times a month
- ☐ Several times a week
- ☐ Every day
- ☐ Several times a day

4.11 Did your child experience social problems because of the smell from accidentally passing stool?

- ☐ No
- ☐ Sometimes; my child didn't miss school, but he/she did not spend the night at other people's places because of the smell
- ☐ Often; my child sometimes missed school and spent less time playing with other children during the day because of the smell

4.12 Is your child's skin red or irritated (rash) **at the moment** because of accidentally passing stool?

- ☐ No
- ☐ Yes

4.13 Was your child given an antidiarrheal medicine to make his/her stool more solid?

- ☐ Never
- ☐ Less than once a month
- ☐ Several times a month
- ☐ Several times a week
- ☐ Every day
- ☐ Several times a day

4.13.1 If your child was given an antidiarrheal medicine, what medicine was this and in what dose?

1. Medicine: _________________	How many times a day: ____<br>Or, how many times a week: ____	Dose: ____ ml/g
2. Medicine: _________________	How many times a day: ____<br>Or, how many times a week: ____	Dose: ____ ml/g
3. Medicine: _________________	How many times a day: ____<br>Or, how many times a week: ____	Dose: ____ ml/g

4.14 Was your child on a special diet or did he/she eat special foods to reduce the risk of accidentally passing stool?

- ☐ Yes, my child eats/drinks: ___________________
- ☐ No

4.15 Did you rinse your child's colon with lukewarm water or a saline solution to avoid him/her accidentally passing stool (via the anus)?

- ☐ Yes, quantity: _____ ml/cc, any additions: ____________
- ☐ No

4.16 Did you ever speak about your child accidentally passing stool? (Multiple answers possible)

- ☐ Yes, with relatives or friends
- ☐ Yes, with the child healthcare centre
- ☐ Yes, with the GP
- ☐ Yes, with a medical specialist
- ☐ Yes, with someone else, namely: ___________________
- ☐ No

Category 5: Urge to defecate

The following questions concern your child's urge to defecate <u>in the past month</u>

5.1 Did your child indicate when he/she felt the urge to defecate or did he/she take the initiative to go to the potty or the toilet?

- ☐ Yes
- ☐ Sometimes
- ☐ No

5.2 On average, how long was your child able to hold his/her stool when he/she felt the urge to defecate?

- ☐ My child was unable to hold his/her stool
- ☐ One minute or less (my child always had to be taken to the toilet at once)
- ☐ At most 5 minutes
- ☐ At most 15 minutes
- ☐ My child never had to hurry

5.3 Was your child able to tell the difference between the urge to pass gass or the urge to defecate?

- ☐ Yes
- ☐ Difficult / sometimes
- ☐ No

Category 6: Urinary continence

The following questions concern your child's bladder control <u>in the last three months</u>

6.1 On average, how often did your child have a wet nappy or how often did he/she have to urinate?

- ☐ Less than three times a day
- ☐ Three to seven times a day
- ☐ More than seven times a day

6.2 Which of the following most closely describes your child's situation? (**Select one answer**)

- ☐ My child is toilet trained for urine
- ☐ My child is being toilet trained for urine
- ☐ My child has not yet started toilet training for urine and still wears a nappy **<u>at all times</u>**

If your child has not yet started toilet training for urine and wears a nappy at all times, please proceed to question 7.1

6.3 When your child had to urinate, was he/she able to release all the urine in one go?

- ☐ Yes, the flow was <u>never</u> interrupted
- ☐ No, the urine <u>sometimes</u> came in trickles
- ☐ No, the urine <u>always</u> came in trickles

6.4 When your child had to urinate, did he/she have to strain?

- ☐ Yes, my child <u>always</u> had to strain when urinating
- ☐ Yes, my child <u>sometimes</u> had to strain when urinating
- ☐ No, my child <u>never</u> had to strain when urinating

6.5 How often did your child accidentally release some urine?

- ☐ Never
- ☐ Minimally once a month
- ☐ Approximately once a week or less
- ☐ Two to three times a week
- ☐ Approximately once a day
- ☐ Several times a day
- ☐ Continuously

If your child never accidentally released urine in the past three months, please proceed to question 6.9

6.6 How much urine did your child usually release?

- ☐ None
- ☐ A little bit (a few drops)
- ☐ Quite a lot (wet underwear)
- ☐ A lot (visible wet spots)

6.7 At what time of the day did your child accidentally release urine?

- ☐ When my child was awake
- ☐ When my child was asleep
- ☐ In both situations

6.8 When did your child accidentally release urine? (Multiple answers possible)

- ☐ Never
- ☐ Before my child could reach the toilet
- ☐ When my child coughed or sneezed
- ☐ When playing/engaging in sports
- ☐ When my child was putting his/her clothes back on after urinating
- ☐ Without apparent trigger
- ☐ Continuously

6.9 How often has you child had a urinary tract infection?

- ☐ Never
- ☐ Once
- ☐ Multiple times, namely ___________ times

6.10 How often was your child treated for a urinary tract infection with antibiotics?

- ☐ Never
- ☐ Once
- ☐ Multiple times, namely ___________ times

umcg

Category 7: Medical history

The following questions concern any conditions or operations that may have affected your child's ability to pass stool

7.1 Did your child undergo one or more of the following operations that may affect defecation? (Multiple answers possible)

- ☐ No, my child never underwent surgery on his/her intestines or anus
- ☐ Removal of a part of the intestine and stitching together of the remaining parts
- ☐ Operation on a fistula between the buttocks near the anus (peri-anal fistula)
- ☐ Operation on a twisted intestine (malrotation)
- ☐ Other, namely: ___________________

Operation to correct a congenital defect such as:

- ☐ Anorectal malformation (anal atresia)
- ☐ Hirschsprung's disease
- ☐ Sacrococcygeal teratoma
- ☐ Other, namely: ___________________

7.2 Did your child ever have an ostomy to help him/her pass stool?

- ☐ Yes, a colostomy
- ☐ Yes, an ileostomy
- ☐ No

7.3 Do your child's stools ever contain blood and/or mucus?

- ☐ Yes
- ☐ No

7.4 Did your child ever have an injury to their anus, medical operations excluded?

- ☐ Yes, namely: ___________________
- ☐ No

7.5 Did or does your child have one of the diseases listed below? (Multiple answers possible)

- ☐ My child did or does not have any of these diseases
- ☐ Irritable bowel syndrome
- ☐ Protrusion of the rectum (rectal prolapse)
- ☐ Necrotizing enterocolitis (NEC)
- ☐ Folding of a section of the bowel into an adjacent section (intussusception)
- ☐ Diabetes mellitus
- ☐ Other neurological conditions (for example, a brain haemorrhage, cerebral palsy)

Congenital defects such as:

- ☐ Anorectal malformation (anal atresia)
- ☐ Hirschsprung's disease
- ☐ Sacrococcygeaal teratoom
- ☐ Spina bifida
- ☐ Other, namely: ___________________

7.6 Does one of the diseases you selected above also occur in your child's family?

- ☐ Yes
- ☐ No
- ☐ Not applicable

7.6.1 If so, which disease and which family member is affected?

Disease: ___________________ Family member: ___________________

Disease: ___________________ Family member: ___________________

Disease: ___________________ Family member: ___________________

Disease: ___________________ Family member: ___________________

7.7 What medicines does your child currently use?
(You do not need to list the above-mentioned laxatives and antidiarrheal medicines)

☐ My child does not use any other medicine
☐ My child uses:

1. Medicine: ___________________	How many times a day: _____	Dose: _____ ml/g
2. Medicine: ___________________	How many times a day: _____	Dose: _____ ml/g
3. Medicine: ___________________	How many times a day: _____	Dose: _____ ml/g
4. Medicine: ___________________	How many times a day: _____	Dose: _____ ml/g
5. Medicine: ___________________	How many times a day: _____	Dose: _____ ml/g
6. Medicine: ___________________	How many times a day: _____	Dose: _____ ml/g

You have reached the end of this questionnaire.

We would like to thank you for your time and for answering the questions.

If there is anything you would like to tell us about your child concerning an aspect that is not or not sufficiently addressed in this questionnaire, please do so below.
