# Supplementary material for: Development and validation of the Early Pediatric Groningen Defecation and Fecal Continence questionnaire
Source: Eur J Pediatr. 2022 Nov 21;182(2):615–23. doi: 10.1007/s00431-022-04714-2 (PMC9899161; doi:10.1007/s00431-022-04714-2)
Supplement: Supplementary file 2 — Supplementary file2 (PDF 91 KB) [file 431_2022_4714_MOESM2_ESM.pdf]

**Supplementary Material 2. Reproducibility of the Early Pediatric Groningen Defecation and Fecal Continence (EP-DeFeC) questionnaire among infants/toddlers and older children**

| | Infants and toddlers (1 month till 4 year old) n = 45 | | | School-aged children (4 years and older) n = 55 | | |
|---|---|---|---|---|---|---|
| **Category** | **Observed agreement (%)** | **Kappa coefficient** | **Interpretation[a]** | **Observed agreement (%)** | **Kappa coefficient** | **Interpretation[a]** |
| Defecation pattern | 70.5 | 0.36 | Fair | 74.1 | 0.28 | Fair |
| Constipation | 74.7 | 0.38 | Fair | 75.6 | 0.43 | Moderate |
| Constipation-related questions | 90.1 | 0.49 | Moderate | 93.2 | 0.77 | Substantial |
| Fecal continence | 63.8 | 0.41 | Moderate | 81.5 | 0.49 | Moderate |
| Urge to defecate | 63.0 | 0.50 | Moderate | 71.5 | 0.37 | Fair |
| Urinary continence | 79.4 | 0.64 | Substantial | 77.6 | 0.53 | Moderate |
| Medical history | 91.1 | 0.61 | Substantial | 86.8 | 0.44 | Moderate |
| **Overall** | **76.1** | **0.49** | **Moderate** | **80.1** | **0.47** | **Moderate** |

[a] Interpretation of kappa coefficients according to Landis & Koch [27]
